# A shape-constrained regression and wild bootstrap framework for reproducible drug synergy testing

**DOI:** 10.64898/2026.02.05.704019

**Published:** 2026-03-30

**Authors:** Amir Asiaee, James P. Long, Samhita Pal, Heather H. Pua, Kevin R. Coombes

**Affiliations:** 1 *Department of Biostatistics, Vanderbilt University Medical Center, Nashville, TN, 37232, USA.; 2 Department of Biostatistics, The University of Texas MD Anderson Cancer Center, Houston, TX, 77030, USA.; 3 Department of Pathology, Microbiology and Immunology, Vanderbilt University Medical Center, Nashville, TN, 37232, USA.; 4 Department of Population Health Sciences, Georgia Cancer Center, Augusta University, Augusta, GA, 30912, USA.

**Keywords:** Drug combinations, synergy, shape-constrained regression, isotonic regression, wild bootstrap, hypothesis testing, reproducibility

## Abstract

High-throughput drug combination screens require methods to identify synergistic pairs, yet widely used synergy scores lack statistical inference and can fail when parametric dose–response fits do not converge. We present SIR (Synergy via Isotonic Regression), a nonparametric framework that defines interaction as deviation from a monotone-additive null, fit by 2D isotonic regression. A degrees-of-freedom-corrected wild bootstrap yields calibrated p-values for each dose–response matrix. On DrugCombDB, SIR interaction surfaces achieve higher replicate concordance (median correlation 0.91 across 1,839 replicate pairs) than all baselines (0.53–0.74), while avoiding Loewe’s 20.9% and ZIP’s 3.6% failure rates. The fitted surface also predicts missing wells (median holdout RMSE 0.040). By replacing heuristic scores with calibrated effect sizes and p-values, SIR enables principled hit calling and error-rate control in large screens.

## 1 Introduction

Drug combinations are a cornerstone of cancer treatment, where monotherapy resistance and an ever-expanding pharmacopeia make rational combination selection critical. Combinations are routinely screened in vitro across dose matrices. Marginal dose-response curves (e.g., Hill or sigmoid models) are often fit to each drug individually to estimate potency and efficacy [1, 2]. These parametric fits are then used to define a “null” expected combination surface under various models of additivity, against which synergy is measured. However, marginal curve fitting can be sensitive to preprocessing and model misfit [3] and can fail to converge (produce no result) on real screening data [4].

Several null models have been proposed for quantifying synergy. Bliss independence [5] assumes that drugs affect cell survival probabilities independently and requires no curve fitting; HSA (highest single agent) simply compares the combination to the more active single agent at each dose pair; Loewe additivity [6, 7] and the Combination Index [8] assume dose equivalence and require fitted marginal curves; and ZIP [4, 9–11] assumes that drugs independently shift each other’s potency without changing curve shape, also requiring marginal fits. Each model encodes a different definition of “no interaction,” so the same dose-response matrix can be called synergistic by one model and antagonistic by another [12–16]. Moreover, all of these approaches produce pointwise scores at each dose pair, which are typically summarized by averaging across the matrix [4, 10]. While standard errors can in principle be computed at individual dose pairs, none of these methods provides a formal statistical test at the matrix level for whether overall interaction is present, nor uncertainty quantification for that conclusion. These limitations are increasingly consequential. Modern machine learning approaches often treat synergy scores as training labels [17–19], but if labels are inconsistent across models or unstable to noise and missingness, predictive models are incentivized to chase idiosyncrasies of the chosen scoring rule rather than reproducible biology. Recent community benchmarks have confirmed that the choice of synergy scoring model is a major source of variation in downstream predictions [19], underscoring the need for stable, statistically grounded alternatives. More fundamentally, without p-values or confidence intervals, it is impossible to distinguish a genuinely interacting combination from one whose synergy score is driven by measurement noise.

We introduce SIR (Synergy via Isotonic Regression), a framework that addresses these shortcomings. SIR replaces parametric curve fitting with monotone regression, a shape constraint that requires only that increasing dose does not decrease effect. Unlike Hill-curve fitting, monotone regression is a convex projection that always has a unique solution for any input data, so SIR never produces undefined outputs. SIR provides a calibrated p-value for each matrix by testing whether the dose-response surface deviates from a monotone-additive null, using a wild bootstrap [20] (a resampling method that generates pseudo-data by randomly flipping residual signs, preserving heteroscedasticity without parametric error assumptions). A global interaction statistic summarizes the entire matrix and, through bootstrap inference, supports reproducible discovery with error-rate control.

## 2 Results

### 2.1 Synergy labels show poor concordance across null models

**Fig. 1:**
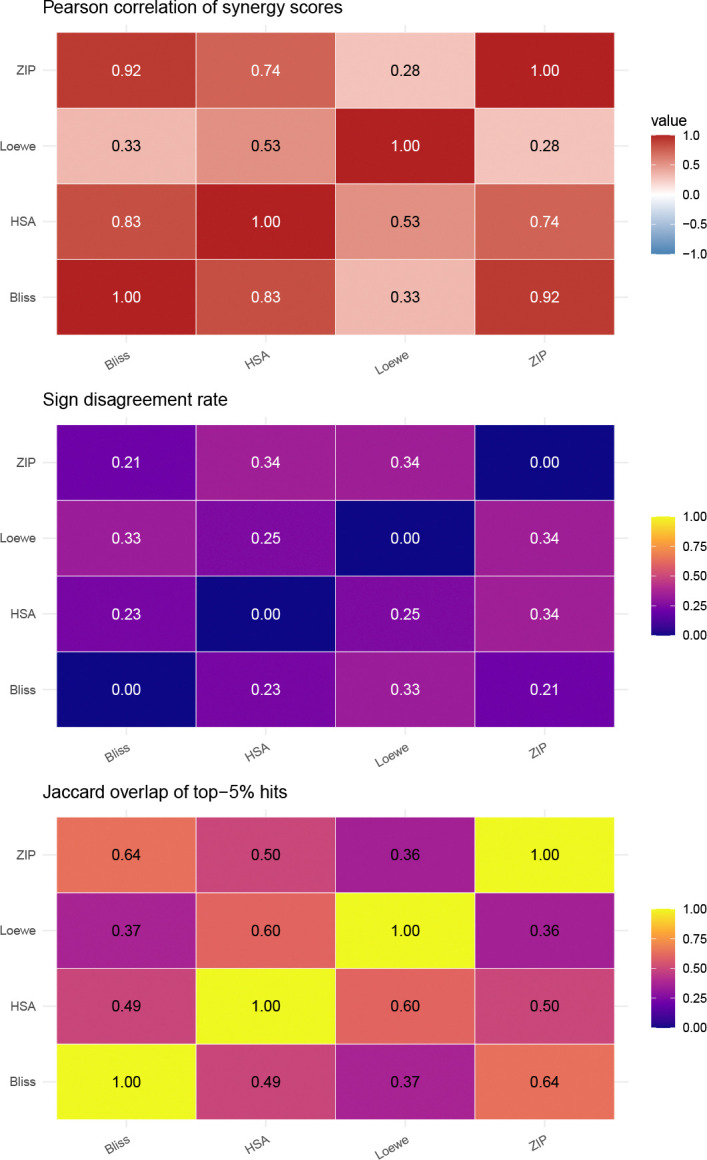
Baseline synergy scores disagree across null models on DrugCombDB (n=391,652 dose–response matrices with valid scores for all four methods). Top: Pearson correlations of matrix-level summary scores. Middle: fraction of matrices where only one method reports positive synergy. Bottom: Jaccard overlap of top 5% synergy calls. Bliss and ZIP correlate highly (r=0.92)), reflecting their shared independence assumptions, whereas Loewe often disagrees with both (r≈0.3), consistent with its fundamentally different dose-equivalence construction. Disagreement extends to hit calling: the Loewe–ZIP Jaccard overlap among top-5% hits is only 0.36, meaning that which drug pairs are prioritized for follow-up depends heavily on the choice of null model. These results motivate a framework that avoids committing to any single null-model definition of additivity.

To quantify disagreement among widely used baselines, we analyzed 391,652 dose–response matrices from DrugCombDB [21] for which all four baseline methods (Bliss, HSA, Loewe, ZIP) returned valid (non-NA) summary scores via SynergyFinder conventions [4]. Each score summarizes a full matrix by averaging pointwise synergy values across dose pairs. These matrix-level scores show strong dependence on the chosen null model: Bliss and ZIP correlate highly (Pearson r=0.92), while Loewe correlates weakly with ZIP (r=0.28) and Bliss (r=0.33) (Fig. 1; pairwise scatterplots in Supplementary Fig. 4). Disagreement is not limited to scaling: for 21–34% of matrices, one method reports synergy (positive score) while the other reports antagonism (Fig. 1). Even “top hit” sets overlap modestly: among the top 5% most synergistic calls, the Loewe–ZIP Jaccard overlap is 0.36 (Fig. 1). These discrepancies imply that “synergy” labels used in downstream analyses, including ML training [17, 18], can vary substantially with the scoring model. The disagreement is not merely academic: a drug pair prioritized as a top hit by one method may rank in the bottom half by another, potentially leading to wasted experimental follow-up or missed therapeutic opportunities.

### 2.2 A shape-constrained definition of interaction

**Fig. 2:**
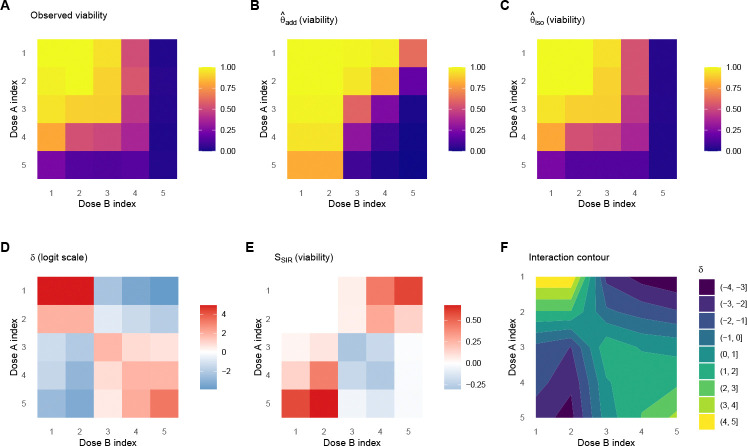
SIR workflow illustrated on a DrugCombDB example (Gemcitabine + Dinaciclib in EFM192B cells; 5 × 5 dose grid). **(A)** Observed viability. **(B)** Monotone-additive null surface ${\hat{\theta }}^{\text{add}}$ (back-transformed to viability): the best-fitting surface under the assumption of no interaction. **(C)** Unconstrained monotone surface ${\hat{\theta }}^{\text{iso}}$ (back-transformed): the best monotone fit without the additivity constraint. **(D)** Interaction surface $\delta ={\hat{\theta }}^{\text{iso}}-{\hat{\theta }}^{\text{add}}$ on the logit scale; negative values (blue) indicate synergy, i.e., the combination kills more than the additive model predicts. **(E)** Effect sizes on the viability scale, $S_{\text{SIR}}={\mathrm{logit}}^{-1}\left({\hat{\theta }}^{\text{add}}\right)-{\mathrm{logit}}^{-1}\left({\hat{\theta }}^{\text{iso}}\right)$. **(F)** Contour map of the interaction surface, showing the spatial pattern of synergy and antagonism across the dose grid. The global interaction test yields p=0.025 (B=200 bootstrap resamples), indicating statistically significant departure from additivity.

We model a drug combination experiment as an I×J grid of responses $Y_{ij}\in [0,1]$ (viability) measured at increasing doses of two drugs. We transform responses to an unconstrained scale, $Z_{ij}=\text{logit}\left(Y_{ij}\right)$, and compute inverse-variance weights from within-cell replicates (Online Methods, Transform and weights). The logit transform maps bounded viability data to ℝ, stabilizes variance near the boundaries, and defines additivity on a scale where equal increments correspond to equal log-odds changes in cell survival. We then fit two nested model classes, both solved as convex quadratic programs (Online Methods, Model classes and estimation): (i) a flexible monotone surface ${\hat{\theta }}^{\text{iso}}\in M$ via 2D isotonic regression (the fitted value at each dose pair is constrained to be non-increasing in each drug’s dose); and (ii) a monotone-additive null surface ${\hat{\theta }}^{\text{add}}\in A\subset M$, $\theta_{ij}=\alpha +u_{i}+v_{j}$, where $u_{i}$ and $v_{i}$ are each constrained to be monotone non-increasing, so that the combined effect is additive with each drug contributing independently. Their difference defines the *interaction surface*,

(1) $$\delta_{ij}={\hat{\theta }}_{ij}^{\text{iso}}-{\hat{\theta }}_{ij}^{\text{add}},$$

interpretable as synergy (for viability) when $\delta_{ij}<0$ (lower viability than additive expectation). Importantly, the interaction surface is not constrained to be monotone; it can take any sign pattern across the grid, capturing spatially heterogeneous interaction (e.g., synergy at some dose pairs and antagonism at others). We summarize the overall strength of interaction across the grid by the *interaction energy* $S^{2}=\sum_{ij}w_{ij}\delta_{ij}^{2}$, a single scalar that measures weighted squared departure from additivity, analogous to a goodness-of-fit statistic comparing the additive model to the unconstrained monotone model (Online Methods, Interaction surface and summaries). We choose $S^{2}$ over alternatives such as the difference in residual sums of squares (SSE_add_ − SSE_iso_) because $S^{2}$ depends only on the interaction surface δ rather than explicitly on residual magnitudes, making it more comparable across datasets in expectation. Note that the interaction signal naturally concentrates at intermediate dose combinations, where one drug is effective enough to reveal synergy or antagonism with the other; at the lowest and highest dose corners, both models predict similar values and δ is typically near zero. A key advantage of this definition is that the null model A is a nested submodel of the alternative M, both within the same monotone function class, so the interaction surface reflects genuine departures from additivity rather than artifacts of comparing models with different structural assumptions. Figure 2 illustrates the SIR workflow on a DrugCombDB example, from observed viability through the fitted surfaces to the interaction map and bootstrap p-value.

### 2.3 Calibrated hypothesis testing by df-corrected wild bootstrap

Existing synergy methods (Bliss, HSA, Loewe, ZIP) return matrix-level synergy scores without p-values, so there is no principled way to assess whether an observed score reflects true interaction or noise, nor to control false-positive rates when screening thousands of drug pairs. SIR addresses this by computing a p-value for each matrix using a wild bootstrap [20, 22] under the monotone-additive null. A standard challenge in bootstrap testing is that residuals from a fitted model underestimate the true noise: because the model was estimated from the same data, it passes closer to the observations than the true underlying dose-response surface would, shrinking the residuals. Bootstrapping with these shrunken residuals produces synthetic datasets that are less variable than real data, biasing the test toward false positives. We correct for this by inflating residuals by a degrees-of-freedom factor before resampling, analogous to the n/(n−p) correction in linear regression variance estimation, where n is the number of observed grid cells and p is the effective degrees of freedom consumed by the null fit (for a typical 5×5 grid with monotone marginals, n=25 and $df_{\text{null}}\approx 9$, giving a ratio of approximately 1.56; Online Methods, Wild bootstrap inference with df correction).

**Fig. 3:**
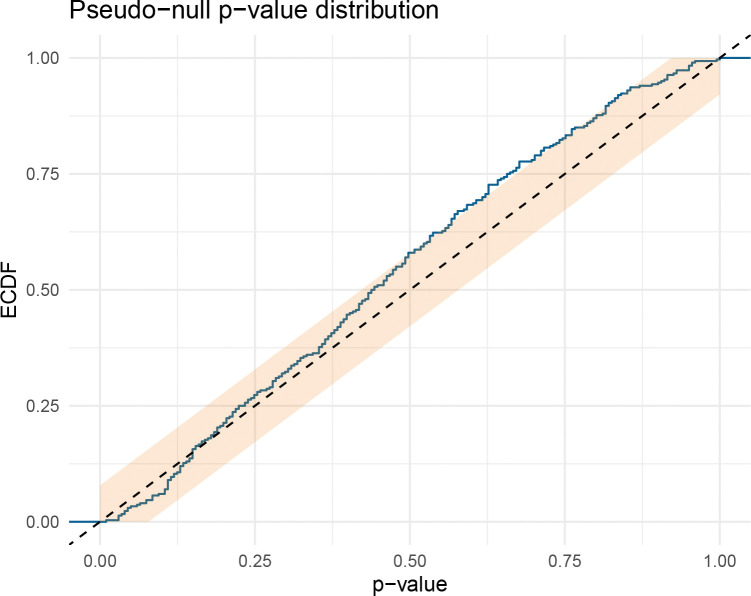
Pseudo-null calibration on DrugCombDB (n=300 randomly selected matrices; B=200). P-values from the df-corrected wild bootstrap when data are generated under the fitted additive null by sign-flipping corrected residuals, so that no true interaction is present by construction. The dashed diagonal is the Uniform(0,1) reference expected for a well-calibrated test; the shaded region is a 95% Dvoretzky–Kiefer–Wolfowitz band. The close agreement with the uniform diagonal demonstrates that SIR’s p-values are trustworthy on real screening data with real noise structure, not just in controlled simulations (Fig. 4).

**Fig. 4:**
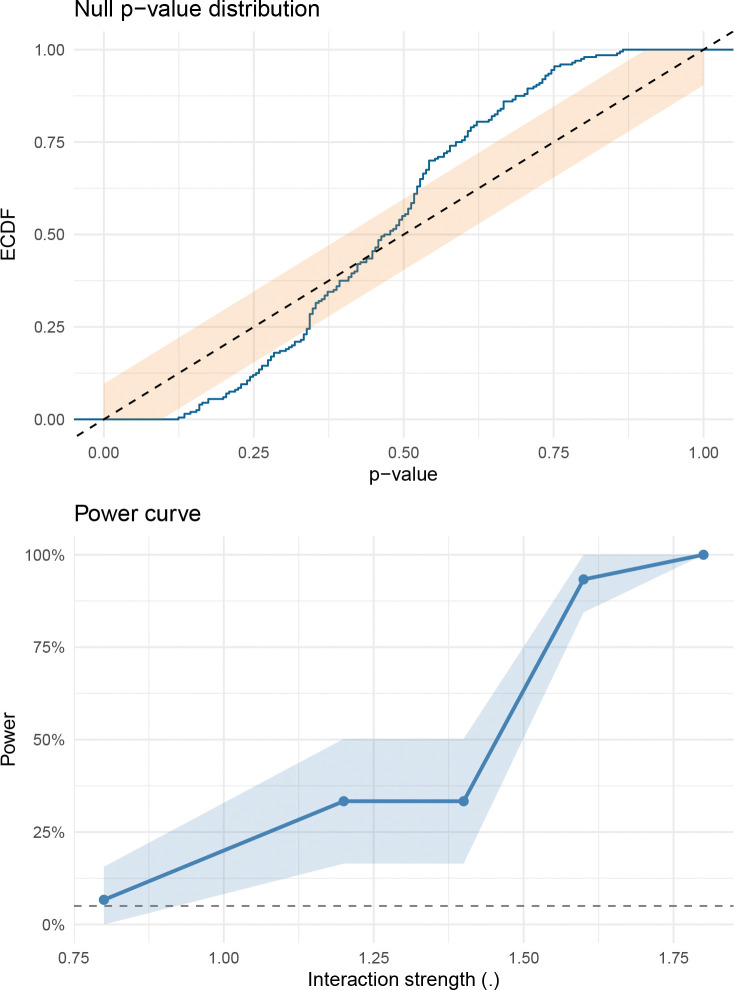
Simulation: calibration and power of the SIR test (8 × 8 grids; σ=0.1 on the logit scale; B=200). Top: empirical cumulative distribution function (ECDF) of p-values under a simulated additive null (n=200 simulations) with 95% Dvoretzky–Kiefer–Wolfowitz band. The CDF closely follows the uniform diagonal, with only minor excursions near the band boundary, confirming that the test does not produce excess false positives. Bottom: power to detect interaction (α=0.05) as interaction strength increases (n=30 simulations per strength). Power rises from near zero at the null to >95% at the strongest departures; the steep transition is typical of power curves and reflects the signal crossing the detection threshold.

To verify that these p-values are well calibrated, we performed a pseudo-null experiment on 300 randomly selected DrugCombDB matrices: for each, we fit the additive null, generated synthetic data by randomly flipping the sign of the corrected residuals (so that no true interaction is present by construction), and reran the full bootstrap test. If the test is properly calibrated, the resulting p-values should follow a uniform distribution, and the false-positive rate at any threshold α should be close to α. Indeed, the empirical p-values closely follow the uniform distribution (Fig. 3), with observed false-positive rates of 3.3% at α=0.05 and 5.7% at α=0.10. At α=0.05, rejecting 3.3% of true nulls (rather than the nominal 5%) means the test is slightly conservative, a desirable property in screening applications where controlling false positives is more important than maximizing detections. We additionally assessed the test’s statistical power in a controlled simulation study. We generated 8 × 8 dose-response grids under a monotone-additive null, then injected interaction of increasing strength by adding a localized bump to the surface (Online Methods, Benchmarks; Supplementary Methods, Simulation study design). Power increases monotonically with interaction strength, rising from near zero at the null to >95% at the strongest departures (Fig. 4). These results confirm that SIR has adequate power to detect biologically meaningful interaction while maintaining calibrated false-positive rates.

### 2.4 Higher reproducibility and zero failures on replicate experiments

**Fig. 5:**
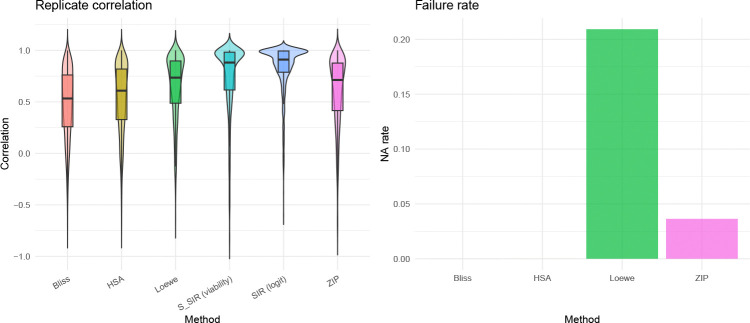
Replicate concordance and failure rates on DrugCombDB (1,839 replicate pairs from 1,209 experiments). Left: replicate correlation of synergy/interaction surfaces for each method. SIR’s interaction surface on the logit scale achieves a median replicate correlation of 0.91; the viability-scale effect sizes $\left(S_{\text{SIR}}\right)$ achieve 0.88. Both are substantially higher than Bliss (0.53), HSA (0.61), ZIP (0.71), or Loewe (0.74), indicating that SIR’s interaction surfaces are more consistent when the same drug pair is measured independently. Right: failure rate (fraction of experiments returning NA/non-finite values). Loewe fails on 20.9% and ZIP on 3.6% of experiments due to marginal curve-fitting failures; SIR never fails because isotonic regression always has a solution.

Reproducible synergy estimates are essential for translating screens into follow-up experiments. If a method assigns different synergy labels to two independent measurements of the same drug pair in the same cell line, its conclusions cannot be trusted. Because these are independent experiments with distinct biological noise, we do not expect identical surfaces, but a reliable method should produce highly correlated interaction patterns. In DrugCombDB, 1,209 unique drug-pair/cell-line combinations were measured in two or more independent experiments, yielding 1,839 replicate pairs (some combinations had three or more experiments, producing multiple pairs). For each replicate pair, we computed the SIR interaction surface δ and the corresponding baseline synergy surfaces, then measured the Pearson correlation between replicate surfaces as a measure of reproducibility (Online Methods, Benchmarks). SIR’s interaction surface δ on the logit scale is substantially more reproducible than all baselines: median replicate correlation is 0.91, compared to 0.53 (Bliss), 0.61 (HSA), 0.74 (Loewe) and 0.71 (ZIP) (Fig. 5). The viability-scale effect sizes $S_{\text{SIR}}$ are similarly reproducible (median 0.88). The higher reproducibility likely reflects the regularizing effect of the monotonicity constraint: by enforcing a biologically motivated shape, SIR suppresses noise-driven fluctuations that inflate variability in unconstrained pointwise scores.

In addition, parametric baselines can be entirely undefined: Loewe and ZIP fail (non-finite output) in 20.9% and 3.6% of experiments, respectively, while SIR succeeds in all cases (Fig. 5). These failures occur when the marginal dose-response curves do not converge or produce degenerate parameter estimates, a problem that is absent in SIR because isotonic regression is a convex projection that always has a unique solution. For large-scale screens where even a small failure rate can affect thousands of matrices, this robustness is a practical necessity.

### 2.5 A generative surface model enables prediction of missing wells

**Fig. 6:**
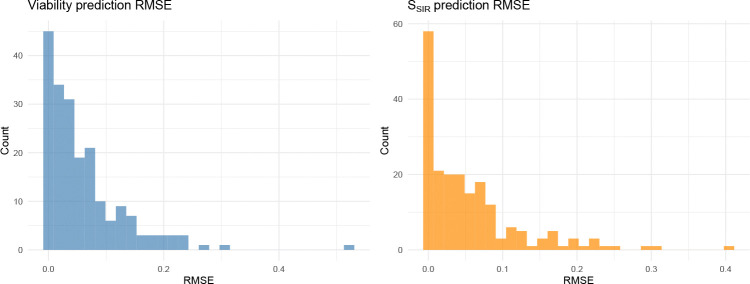
Prediction accuracy under missingness on DrugCombDB (n=200 matrices, 20% interior wells held out). Left: holdout RMSE for viability predictions. Right: holdout RMSE for the SIR synergy summary $S_{\text{SIR}}$. Median prediction error is 0.040 in viability units and 0.035 for $S_{\text{SIR}}$, indicating that the monotone surface captures the underlying dose-response relationship well enough to impute missing measurements. This capability is unique to SIR: existing synergy scores are computed pointwise and cannot predict unobserved dose pairs.

Drug combination matrices are often incomplete due to plate layout constraints, assay failures, or adaptive designs. Standard synergy scores cannot handle missing entries because they are computed independently at each dose pair; a missing well means a missing score. Because SIR fits an explicit monotone surface to all available data, it naturally predicts unobserved dose pairs by evaluating ${\hat{\theta }}^{\text{iso}}$ at the missing coordinates and transforming back to viability. In a holdout benchmark on 200 DrugCombDB matrices, we withheld 20% of interior wells and predicted their viabilities from the remaining data. Predictions are accurate (median RMSE 0.040 in viability units), and the induced synergy summaries are similarly stable (median RMSE 0.035 for the cellwise $S_{\text{SIR},ij}$ values) (Fig. 6). This capability is particularly relevant for adaptive screening designs, where only a subset of dose pairs may be measured in a first pass, and the fitted surface can guide selection of follow-up wells. It also means that SIR can produce synergy summaries even for matrices with irregular or non-rectangular dose layouts, a setting where pointwise baselines would require imputation or exclusion of missing entries.

## 3 Discussion

SIR is a statistical framework for drug combination interaction that replaces fragile parametric curve fitting with shape-constrained regression and supplies calibrated p-values by a df-corrected wild bootstrap. The method addresses three recurrent problems in synergy analysis (Table 1). First, baseline null models disagree substantially on the same data (Fig. 1), implying that “synergy” labels are not a stable ground truth [12, 14]. Second, baselines that rely on parametric marginal models (Loewe, ZIP [9]) can fail on real matrices, whereas SIR’s isotonic regression [23] always yields a feasible monotone fit (Fig. 5). Third, the absence of uncertainty quantification in common scoring approaches, including the widely used Combination Index [8, 24], prevents error-rate control and principled aggregation across experiments.

These issues are relevant for machine-learning-guided combination discovery. ML pipelines that predict synergy scores [17–19] inherit label noise when those scores are inconsistent across models or undefined due to fit failures. SIR may provide more defensible training labels by returning calibrated effect sizes and p-values rather than heuristic scores, though a direct comparison of downstream ML performance remains future work.

### Relationship to other frameworks.

MuSyC [3] takes a complementary approach: it fits a parametric multi-parameter surface to disentangle efficacy, potency, and cooperativity synergy. By contrast, SIR avoids parametric assumptions entirely and focuses on calibrated statistical testing of whether any interaction is present, rather than decomposing its mechanistic origin. Response surface approaches [13] and software tools such as Combenefit [11] provide visualization and quantification of synergy using Loewe, Bliss, or HSA, but inherit the limitations of those null models and do not provide calibrated statistical tests. The two frameworks (SIR and MuSyC) address different questions and could be used in sequence: SIR to identify interacting pairs with controlled error rates, followed by parametric modeling (MuSyC or others) to characterize the nature of confirmed interactions.

### Direction of interaction and multiplicity.

The global interaction energy $S^{2}$ is inherently two-sided: it tests whether the dose-response surface deviates from monotone additivity anywhere, without committing to a single sign across the grid. After rejecting the global null, users may summarize direction using directional energies (Supplementary Methods, Global and directional summaries) and, if desired, test whether the interaction is predominantly synergistic or antagonistic. Because this directional test is only performed after the global test has already confirmed that interaction exists, it does not require a separate multiple-testing correction: the global test acts as a gatekeeper that controls the overall false-positive rate.

### Monotonicity as a minimal assumption.

Monotonicity can fail in cases such as hormesis or biphasic responses [15]. Rather than assuming a parametric form, we treat monotonicity as a regularizing constraint, a weak assumption that stabilizes estimation on sparse, noisy grids without imposing a specific functional form. Dataset-scale diagnostics on DrugCombDB indicate that most apparent monotonicity violations are small in magnitude and consistent with noise (Supplementary Fig. 1), supporting monotone regression as a pragmatic default. When strong non-monotonicity is suspected, diagnostics can flag affected matrices for alternative modeling.

### Interaction at dose extremes.

The interaction surface δ naturally concentrates at intermediate dose combinations: at the lowest doses, neither drug is effective and both models predict viability near one (a ceiling effect); at the highest doses, both drugs independently drive viability toward zero, leaving little room for additional combinatorial effect (a floor effect). This is pharmacologically expected and does not bias the global test, because $S^{2}$ sums over all grid cells and near-zero δ values at the corners contribute negligibly. The logit transform partially mitigates the compression by stretching both tails, but the floor effect persists in practice. Users interested in dose-specific synergy should examine the interaction surface directly rather than relying solely on the global test.

### Choice of transform.

The null hypothesis of monotone additivity is defined on the scale determined by the response transform. Because different transforms (logit, identity, asinh) impose different geometries, inference results can depend on this choice, a property shared by all parametric and semiparametric interaction tests. We recommend the logit link as a principled default for bounded viability data: it maps [0,1] to ℝ, stabilizes variance near the boundaries, and defines additivity on a scale where equal increments correspond to equal log-odds changes. When the appropriate scale is uncertain, we recommend reporting results under two or more transforms as a sensitivity check (Supplementary Note 2).

### Limitations.

SIR tests for departure from monotone additivity but does not decompose the interaction into mechanistic components (e.g., potency versus efficacy synergy, as in MuSyC [3]). The monotone-additive null treats each drug’s contribution as a fixed monotone function, which may be overly flexible for low-dimensional grids (e.g., 3 × 3) where few data points are available for estimation; in such settings, the test may have limited power. The wild bootstrap assumes that errors are independent across grid cells, which could be violated if systematic plate effects or spatial correlations are present. Finally, while the logit transform is a principled default, the choice of transform affects the null hypothesis and therefore the results, and there is no universally correct scale for defining additivity.

### Practical guidance.

For exploratory screens with thousands of matrices, we recommend B=200 bootstrap resamples with Benjamini–Hochberg FDR correction on the resulting p-values. For confirmatory studies focused on a small number of candidate combinations, B should be increased to 1,000 or more to achieve finer p-value resolution. Grid sizes of 4 × 4 or larger provide sufficient degrees of freedom for the monotone-additive fit; smaller grids may benefit from reduced models. When within-cell replicates are available, inverse-variance weighting improves efficiency by down-weighting noisy dose pairs. SIR’s fitted surfaces and interaction maps can be visualized alongside the scalar p-value to provide spatial insight into where on the dose grid interaction is strongest.

### Outlook.

The framework readily extends to alternative response transforms, weighted designs, and different global statistics. SIR is, to our knowledge, the first synergy-testing framework that combines isotonic regression, a monotone-additive null projection, and calibrated bootstrap p-values for drug combination screens. More broadly, replacing heuristic scores with statistically grounded estimators can help reconcile the fragmented landscape of synergy models [13, 15, 25] by enabling calibrated discovery and reproducible benchmarking. As a generalizability check, we replicated the baseline disagreement and pseudo-null calibration analyses on NCI-ALMANAC [26], an independent large-scale combination screen; both findings held (Supplementary Figs. 2–3). Looking forward, SIR could be extended to higher-order combinations (three or more drugs) by defining monotone-additive null models on higher-dimensional dose grids, though computational cost and the curse of dimensionality would need to be addressed. Integration with downstream machine learning pipelines, where SIR’s calibrated p-values and interaction surfaces replace heuristic synergy scores as training labels, is another promising direction.

## 4 Online Methods

### 4.1 Data sources and preprocessing

We evaluated the method primarily on DrugCombDB [21], a curated database of drug combination experiments with dose matrices across many drugs and cell lines. We used viability responses scaled to [0,1] and standardized doses to a common unit when available. DrugCombDB provides baseline synergy scores for Bliss, HSA, Loewe and ZIP via SynergyFinder conventions [4]; we used these for baseline disagreement analyses.

### 4.2 Transform and weights

Let $Y_{ij}\in [0,1]$ denote viability at dose pair (i,j). We transform responses to $Z_{ij}=\text{logit}\left(Y_{ij}\right)$, clamping $Y_{ij}$ to [ϵ,1−ϵ] with $\epsilon =10^{-6}$ to avoid infinities. We use the logit link because viability is bounded in [0, 1] and the logit stabilizes variance near the boundaries; alternative transforms (identity, asinh, log) are explored in Supplementary Note 2. When replicate measurements are available, we compute inverse-variance weights

(2) $$w_{ij}=\frac{m_{ij}}{\max\left(s_{ij}^{2},\tau \right)},$$

where $m_{ij}$ is the number of replicates, $s_{ij}^{2}$ is the sample variance, and $\tau =10^{-6}$ is a variance floor that prevents degenerate weights. Weights are Winsorized at the 99th percentile to limit the influence of extremely low-variance cells. When no replicates are available $\left(m_{ij}=1\right)$, all weights default to 1/τ (i.e., uniform weighting).

### 4.3 Model classes and estimation

Let M denote the set of monotone surfaces on an I×J grid (non-increasing in each coordinate for viability). The isotonic estimator is the weighted least-squares projection

(3) $${\hat{\theta }}^{\text{iso}}=\arg\underset{\theta \in M}{\min}\underset{i,j}{\sum }w_{ij}{\left(Z_{ij}-\theta_{ij}\right)}^{2}.$$

Let A⊂M denote the monotone-additive class $\theta_{ij}=\alpha +u_{i}+v_{j}$, with u and v constrained to be monotone in the same direction and with identifiability constraints $\left(u_{1}=0,v_{1}=0\right)$ to fix the intercept and prevent trading constants between α, u, and v. We compute ${\hat{\theta }}^{\text{add}}$ as the weighted least-squares projection onto A. Both problems are solved as convex quadratic programs with linear inequality constraints using OSQP [27].

### 4.4 Interaction surface and summaries

The interaction surface $\delta_{ij}={\hat{\theta }}_{ij}^{\text{iso}}-{\hat{\theta }}_{ij}^{\text{add}}$ is defined on the logit scale. To report effect sizes in interpretable viability units, we back-transform each surface and take their difference:

(4) $$S_{\text{SIR},ij}={\mathrm{logit}}^{-1}\left({\hat{\theta }}_{ij}^{\text{add}}\right)-{\mathrm{logit}}^{-1}\left({\hat{\theta }}_{ij}^{\text{iso}}\right),$$

so that positive values of $S_{\text{SIR}}$ indicate synergy (the combination kills more than the additive prediction). Global interaction strength is summarized by the interaction energy $S^{2}=\sum_{ij}w_{ij}\delta_{ij}^{2}$. For the bootstrap test, we use the normalized variant $T=S^{2}/\sum_{ij}w_{ij}$, which adjusts for grid size and weighting.

### 4.5 Wild bootstrap inference with df correction

To test the null hypothesis of monotone additivity, we use a Rademacher wild bootstrap [20]. We fit the null model ${\hat{\theta }}^{\text{add}}$ and compute residuals $r_{ij}=Z_{ij}-{\hat{\theta }}_{ij}^{\text{add}}$. Because the fitted null absorbs some noise, residuals underestimate the true error variance. We correct for this by inflating residuals:

(5) $${\tilde{r}}_{ij}=r_{ij}\sqrt{\frac{n_{\text{eff}}}{n_{\text{eff}}-df_{\text{null}}}},$$

where $n_{\text{eff}}$ is the number of grid cells with finite, positively weighted observations, and $df_{\text{null}}$ is the effective degrees of freedom consumed by the monotone-additive fit. Unlike linear regression, where df equals the fixed number of parameters, isotonic regression pools adjacent dose levels that violate monotonicity into tied groups, so its effective df depends on the data: if the observed marginal responses are already monotone, each dose level retains its own fitted value and df is large; if they are highly non-monotone, many levels are pooled and df is small. We approximate $df_{\text{null}}$ by counting the number of distinct fitted levels in the monotone main effects $\hat{u}$ and $\hat{v}$ (see Supplementary Methods, Degrees-of-freedom correction in the wild bootstrap). We then generate B bootstrap datasets $Z_{ij}^{⋆}={\hat{\theta }}_{ij}^{\text{add}}+\xi_{ij}{\tilde{r}}_{ij}$, where $\xi_{ij}\in \{-1,+1\}$ are independent Rademacher random variables [20, 22], refit both models on each $Z^{⋆}$, and recompute the test statistic $T_{b}^{⋆}$. The p-value is

(6) $$p=\frac{1+\#\left\{T_{b}^{⋆}\geq T_{\text{obs}}\right\}}{B+1}.$$

The default B=200 provides adequate resolution for exploratory screens (p-value increments of ≈0.005); for confirmatory analyses requiring stringent thresholds (e.g., α<0.01 after multiple-testing correction), B should be increased to 1,000–5,000. On a single core, the full test takes ≈0.9s per 4 × 4 matrix and ≈1.5s per 10 × 10 matrix with B=200; a 400,000-matrix screen completes in ≈14 hours on 8 cores.

### 4.6 Multiple testing in screens

When applying the test across a screen of M matrices, standard false-discovery-rate procedures (e.g., Benjamini–Hochberg [28]) can be applied directly to the per-matrix p-values. Users should note that the smallest achievable p-value is 1/(B+1); for large screens where the FDR threshold may require very small p-values, increasing B is recommended. Stratifying by cell line or plate before applying FDR correction can improve power, because matrices within the same cell line or plate tend to share similar noise levels, making the p-value distribution within each stratum more homogeneous.

### 4.7 Benchmarks

**Baseline disagreement:** We computed pairwise correlations, sign disagreement rates, and top-hit overlaps across baseline scores on DrugCombDB matrices with valid (non-NA) Bliss, HSA, Loewe and ZIP scores (391,652 matrices). **Replicate concordance:** We sampled repeated experiments for the same drug pair and cell line, computed synergy/interaction surfaces for each replicate experiment, and evaluated replicate correlations and failure rates. **Missingness prediction:** For each of 200 matrices, we held out 20% of interior wells, fit the SIR model on the remaining wells, and evaluated RMSE on held-out viabilities and derived synergy summaries. **Pseudo-null calibration:** We generated pseudo-null data by sign-flipping df-corrected residuals from the fitted null and recomputed bootstrap p-values. **Simulation:** We constructed monotone-additive null surfaces on 8×8 grids (intercept plus monotone row and column effects), then injected interaction by adding a localized bump projected onto the monotone cone to ensure model-consistent alternatives (see Supplementary Methods, Simulation study design). Gaussian noise (σ=0.1 on the logit scale) was added to generate observed data. Under the null (no injected interaction), we evaluated Type I error calibration by running n=200 simulations and checking that p-values are uniformly distributed (Fig. 4, top). Under the alternative (injected interaction at five positive strengths), we evaluated power by running n=30 simulations per strength and measuring rejection rate at α=0.05 (Fig. 4, bottom). All simulations used B=200 bootstrap resamples.

## Supplementary Material

Supplement 1

## Figures and Tables

**Table 1:** Capabilities of SIR versus common baselines.

Capability	SIR	Bliss	HSA	Loewe	ZIP
Statistical test (calibrated p-value)	✓	–	–	–	–
Predicts missing wells	✓	–	–	–	–
Shape-constrained regularization	✓	–	–	–	–
No fit failures on real data	✓	✓	✓	–	–

Null assumption	Monotone	Indep.	Max effect	Dose equiv.	Indep. + curve fit

✓ = present; – = absent. Baselines provide pointwise scores without uncertainty; some require parametric curve fitting, which can fail on real data.

## Data Availability

DrugCombDB data are publicly available at http://drugcombdb.denglab.org/ [21]. NCI-ALMANAC data are available from the National Cancer Institute at https://wiki.nci.nih.gov/display/NCIDTPdata/NCI-ALMANAC. Download instructions, checksums, and file placement details are provided in the repository README.

## 4.8 Code availability

The SIR R package and all analysis scripts are available at https://github.com/AsiaeeLab/SIR (Supplementary Note 1). The repository includes source code, configuration files, experiment notebooks, and instructions to reproduce all figures and tables in this manuscript.
