# Supplementary material for: A shape-constrained regression and wild bootstrap framework for reproducible drug synergy testing
Source: bioRxiv. 2026 Mar 30:2026.02.05.704019. Preprint. [Version 2] doi: 10.64898/2026.02.05.704019 (PMC13060067; doi:10.64898/2026.02.05.704019)
Supplement: Supplement 1 [file NIHPP2026.02.05.704019v2-supplement-1.pdf]

bioRxiv preprint doi: https://doi.org/10.64898/2026.02.05.704019; this version posted March 30, 2026. The copyright holder for this preprint (which was not certified by peer review) is the author/funder, who has granted bioRxiv a license to display the preprint in perpetuity. It is made available under aCC-BY 4.0 International license.

Supplementary Information for: A shape-constrained regression and wild bootstrap framework for reproducible drug synergy testing

Amir Asiaee[1*], James P. Long[2], Samhita Pal[1], Heather H. Pua[3], Kevin R. Coombes[4]

[1*]Department of Biostatistics, Vanderbilt University Medical Center, Nashville, TN, 37232, USA.
[2]Department of Biostatistics, The University of Texas MD Anderson Cancer Center, Houston, TX, 77030, USA.
[3]Department of Pathology, Microbiology and Immunology, Vanderbilt University Medical Center, Nashville, TN, 37232, USA.
[4]Department of Population Health Sciences, Georgia Cancer Center, Augusta University, Augusta, GA, 30912, USA.

*Corresponding author(s). E-mail(s): amir.asiaeetaheri@vumc.org;

Supplementary Methods

1 Model-consistent definition of interaction

This section provides the formal definitions underlying the SIR interaction surface introduced in the main text. The key idea is that both the null model (no interaction) and the alternative model (unrestricted interaction) are defined within the same class of monotone functions, so that any detected interaction reflects a genuine departure from additivity rather than an artifact of comparing models with different structural assumptions.

Let $Z \in \mathbb{R}^{I\times J}$ denote transformed responses on an $I \times J$ dose grid and $w_{ij} \geq 0$ denote weights. Let $\mathcal{M}$ be the set of monotone surfaces (for viability, non-increasing in each coordinate) and let $\mathcal{A} \subset \mathcal{M}$ be the monotone-additive class $\theta_{ij} = \alpha + u_i + v_j$, where u_i and v_j are each constrained to be monotone non-increasing, with identifiability constraints $u_1 = 0$ and $v_1 = 0$ to prevent trading constants between the intercept α

bioRxiv preprint doi: https://doi.org/10.64898/2026.02.05.704019; this version posted March 30, 2026. The copyright holder for this preprint (which was not certified by peer review) is the author/funder, who has granted bioRxiv a license to display the preprint in perpetuity. It is made available under aCC-BY 4.0 International license.

and the marginal effects. We define the isotonic and additive estimators as weighted projections:

$$\hat{\theta}^{\text{iso}} = \arg\min_{\theta \in \mathcal{M}} \sum_{i,j} w_{ij}(Z_{ij} - \theta_{ij})^2, \tag{1}$$

$$\hat{\theta}^{\text{add}} = \arg\min_{\theta \in \mathcal{A}} \sum_{i,j} w_{ij}(Z_{ij} - \theta_{ij})^2. \tag{2}$$

The interaction surface is

$$\delta = \hat{\theta}^{\text{iso}} - \hat{\theta}^{\text{add}}. \tag{3}$$

This definition is *model-consistent*: interaction is defined as the component of the best monotone fit that cannot be explained by the best monotone-additive fit, within the same constrained geometry.

Why the weighted mean interaction is zero.

Both $\hat{\theta}^{\text{iso}}$ and $\hat{\theta}^{\text{add}}$ are translation-invariant projections: if $\theta \in \mathcal{M}$ (or $\mathcal{A}$), then $\theta + c\mathbf{1}$ is feasible for any constant c. With a squared-error objective, translation invariance implies that both projections preserve the weighted mean of Z, and therefore $\sum_{ij} w_{ij}\delta_{ij} = 0$. Consequently, interaction can be mixed-sign even for strongly synergistic combinations; directional summaries should therefore avoid relying on the signed mean of δ.

2 Global and directional summaries

Global interaction energy.

We summarize interaction magnitude with the weighted energy

$$S^2 = \sum_{i,j} w_{ij}\delta_{ij}^2, \tag{4}$$

and report either the normalized mean energy $S^2/\sum w$ or a studentized version $S^2/\text{SSE}_{\text{add}}$ for inference, where $\text{SSE}_{\text{add}} = \sum_{i,j} w_{ij}(Z_{ij} - \hat{\theta}_{ij}^{\text{add}})^2$. The default test statistic throughout this paper is $T = S^2/\sum_{ij} w_{ij}$; the studentized alternative is noted for completeness but is not used in any reported results.

Directional energies and a bounded direction index.

For viability, synergy corresponds to $\delta_{ij} < 0$. Define one-sided energies

$$S_-^2 = \sum_{i,j} w_{ij} \max(-\delta_{ij}, 0)^2, \qquad S_+^2 = \sum_{i,j} w_{ij} \max(\delta_{ij}, 0)^2. \tag{5}$$

We summarize direction by the bounded index

$$I_{\text{syn}} = \frac{S_-^2 - S_+^2}{S_-^2 + S_+^2} \in [-1, 1], \tag{6}$$

bioRxiv preprint doi: https://doi.org/10.64898/2026.02.05.704019; this version posted March 30, 2026. The copyright holder for this preprint (which was not certified by peer review) is the author/funder, who has granted bioRxiv a license to display the preprint in perpetuity. It is made available under aCC-BY 4.0 International license.

where values near $+1$ indicate predominantly synergy-like interaction (negative deviations for viability), values near -1 indicate antagonism-like interaction, and values near 0 indicate mixed-sign interaction.

Sequential inference and multiplicity.

The global test based on S^2 (or $S^2/\text{SSE}_{\text{add}}$) answers "does interaction exist?" and is inherently two-sided. Directional inference can be performed after rejecting the global null using a step-down procedure: first test interaction at level α; only if rejected, test direction using a pre-specified directional statistic (e.g., I_{syn} or $S_-^2 - S_+^2$) with an appropriate bootstrap reference. Because directional testing is only performed after the global test has confirmed that interaction exists, the global test acts as a gatekeeper: it controls the overall false-positive rate without requiring a separate multiple-testing correction for the directional step. This is analogous to only examining individual regression coefficients after a global F-test has rejected the null that all coefficients are zero.

3 Degrees-of-freedom correction in the wild bootstrap

The wild bootstrap generates pseudo-data under the fitted null:

$$Z_{ij}^{\star} = \hat{\theta}_{ij}^{\text{add}} + \xi_{ij}\tilde{r}_{ij}, \qquad \xi_{ij} \in \{-1, +1\} \text{ i.i.d.}, \qquad \tilde{r}_{ij} = r_{ij} \cdot s, \tag{7}$$

where $r_{ij} = Z_{ij} - \hat{\theta}_{ij}^{\text{add}}$ are null residuals and s is a residual scaling factor. Because $\hat{\theta}^{\text{add}}$ is estimated from the same data, residuals are shrunk relative to the true noise variance. We therefore inflate residuals by a degrees-of-freedom (df) factor

$$s = \sqrt{\frac{n_{\text{eff}}}{n_{\text{eff}} - df_{\text{null}}}}, \tag{8}$$

where n_{eff} is the number of finite, positively weighted grid cells and df_{null} is the effective degrees of freedom consumed by the monotone-additive fit.

Unlike linear regression, where degrees of freedom equals the fixed number of parameters, isotonic regression pools adjacent dose levels that violate monotonicity into tied groups, so its effective df depends on the data. If the observed marginal responses are already monotone, each dose level retains its own fitted value and df is large; if they are highly non-monotone, many levels are pooled and df is small. We approximate df_{null} by counting the number of distinct fitted levels in the monotone main effects $\hat{u}$ and $\hat{v}$. This approximation is analogous to counting the number of free parameters in a piecewise-constant fit: each group of dose levels that share the same fitted value acts as one free parameter, so the total df is approximately the number of such groups across both margins. See [1] for wild bootstrap foundations.

4 Simulation study design

We constructed simulated dose-response data as follows.

bioRxiv preprint doi: https://doi.org/10.64898/2026.02.05.704019; this version posted March 30, 2026. The copyright holder for this preprint (which was not certified by peer review) is the author/funder, who has granted bioRxiv a license to display the preprint in perpetuity. It is made available under aCC-BY 4.0 International license.

Null surface generation.

For each simulation replicate, we drew monotone row effects $u_1 \geq u_2 \geq \cdots \geq u_8$ and column effects $v_1 \geq v_2 \geq \cdots \geq v_8$ independently from standard normal distributions and then sorted them to enforce monotonicity. An intercept α was drawn from Uniform$(1, 3)$ to place the surface at biologically plausible viability levels. The null surface is $\theta_{ij}^{\text{add}} = \alpha + u_i + v_j$ on the logit scale.

Interaction injection.

To generate alternatives, we added a localized bump centered at the middle of the 8×8 grid. The bump amplitude at each cell is $A \cdot \exp\big(-\|(i, j) - \text{center}\|^2/(2\sigma^2)\big)$, where A is the interaction strength parameter, center $= (4.5, 4.5)$, and $\sigma = 2$. The bump is then projected onto the monotone cone $\mathcal{M}$ to ensure that the resulting surface remains feasible under the alternative model; this makes the alternative *model-consistent* (interaction is detectable by $\delta = \hat{\theta}^{\text{iso}} - \hat{\theta}^{\text{add}}$).

Observed data.

Gaussian noise $\varepsilon_{ij} \sim \mathcal{N}(0, \sigma_\varepsilon^2)$ with $\sigma_\varepsilon = 0.1$ (on the logit scale) was added to each cell of the surface to generate observed Z_{ij}. No replicates were simulated ($m_{ij} = 1$, uniform weights).

Simulation grid.

Interaction strengths $A \in \{0, 0.8, 1.2, 1.4, 1.6, 1.8\}$ were used. At $A = 0$ (the null), we ran $n = 200$ independent replicate simulations to obtain a precise empirical CDF for the Type I error check (Fig. 4, top panel, in the main text). At each of the five positive strengths ($A > 0$), we ran $n = 30$ replicates and measured the rejection rate at $\alpha = 0.05$ to estimate power (Fig. 4, bottom panel). All simulations used $B = 200$ bootstrap resamples.

5 Dataset-scale monotonicity diagnostics

SIR assumes that the dose-response surface is monotone (non-increasing in each drug's dose for viability endpoints). This assumption is biologically motivated by the expectation that higher drug concentrations should not decrease killing, but it may be violated in practice by measurement noise or genuine non-monotone phenomena such as hormesis. To assess how often and how severely monotonicity is violated in real screening data, we computed diagnostic statistics on DrugCombDB.

Diagnostic definition.

For each matrix, we check every pair of adjacent dose levels along each drug's axis (holding the other drug's dose fixed) and record whether viability increases rather than decreases with dose. For a 5×5 grid, there are $(5 - 1) \times 5 = 20$ such adjacent comparisons per axis; for a 4×4 grid, $(4 - 1) \times 4 = 12$. We report the fraction of these comparisons that violate monotonicity along drug A (f_A) and drug B (f_B), as well as the maximum magnitude of any violation (m) in viability units. We summarize each matrix by $f_{\text{joint}} = (f_A + f_B)/2$ and $m = \max(m_A, m_B)$.

bioRxiv preprint doi: https://doi.org/10.64898/2026.02.05.704019; this version posted March 30, 2026. The copyright holder for this preprint (which was not certified by peer review) is the author/funder, who has granted bioRxiv a license to display the preprint in perpetuity. It is made available under aCC-BY 4.0 International license.

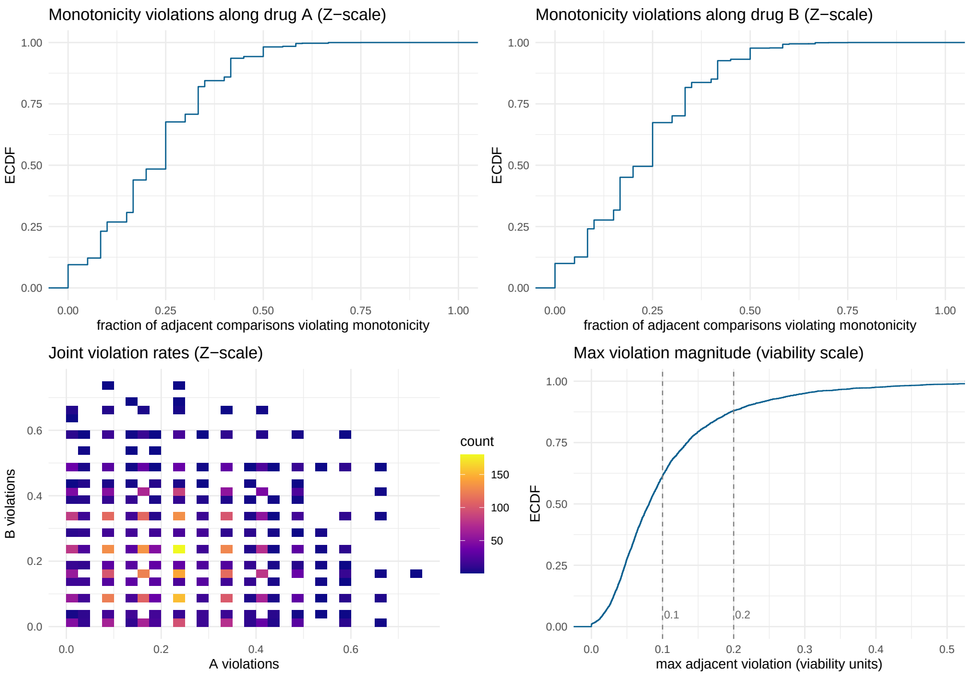

Supplementary Fig. 1 Monotonicity violation frequency and magnitude on DrugCombDB (5,000 randomly sampled matrices). Top row: empirical CDFs of the fraction of adjacent dose comparisons that violate monotonicity along each drug's dose axis. Bottom left: joint distribution of violation fractions across both axes. Bottom right: empirical CDF of the maximum adjacent violation magnitude on the viability scale; dashed lines at 0.1 and 0.2 viability units for reference. Violations are frequent (median fraction ≈0.25 per axis) but small in magnitude (median maximum violation 0.081; 90th percentile 0.219), consistent with measurement noise rather than systematic non-monotone biology. These results support monotone regression as a pragmatic default; the small fraction of matrices with large violations (<5% exceed 0.3) can be flagged for inspection or alternative modeling.

Empirical results on DrugCombDB.

On a random sample of 5,000 DrugCombDB matrices (3,538 of size 4×4 and 1,462 of size 5×5), the median violation fraction was $f_A = f_B = 0.25$, meaning roughly one in four adjacent comparisons shows a reversal. In concrete terms, a typical 5×5 matrix has about 5 violations out of 20 adjacent pairs per axis, and a typical 4×4 matrix about 3 out of 12. Crucially, these violations are small in magnitude: the median maximum adjacent violation was only 0.081 in viability units (90th percentile 0.219; 95th percentile 0.298), and 61.2% (87.9%) of matrices had maximum adjacent violation ≤ 0.1 (≤ 0.2). This pattern is consistent with measurement noise rather than systematic non-monotone biology: genuine hormesis or biphasic effects would produce large, consistent reversals across multiple dose pairs, not the scattered small fluctuations observed here.

bioRxiv preprint doi: https://doi.org/10.64898/2026.02.05.704019; this version posted March 30, 2026. The copyright holder for this preprint (which was not certified by peer review) is the author/funder, who has granted bioRxiv a license to display the preprint in perpetuity. It is made available under aCC-BY 4.0 International license.

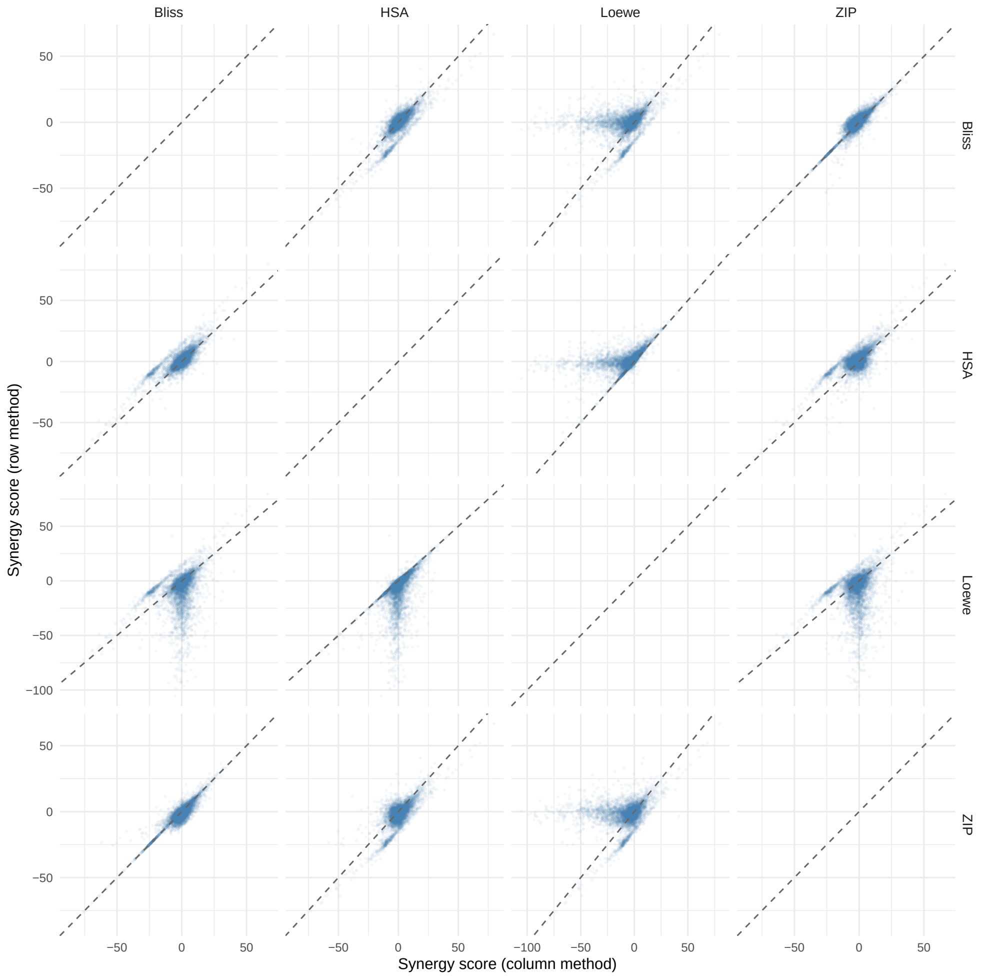

Supplementary Fig. 2 Pairwise scatterplots of baseline synergy scores on DrugCombDB (5,000 randomly subsampled matrices). Each panel shows one method's matrix-level summary score against another; the dashed diagonal is the identity line. The near-linear Bliss–ZIP relationship (top right) confirms their high correlation ($r = 0.92$), while the Loewe–ZIP and Loewe–Bliss panels reveal broad clouds with many sign-discordant points (opposite quadrants), explaining the low correlations ($r \approx 0.3$) and high sign disagreement rates reported in Fig. 1 of the main text.

6 Supplementary Discussion: baseline disagreement metrics

To quantify the extent to which different synergy scoring methods agree on which drug combinations are synergistic, we computed three complementary metrics across all DrugCombDB dose-response matrices with valid (non-NA) scores for all four baseline methods.

bioRxiv preprint doi: https://doi.org/10.64898/2026.02.05.704019; this version posted March 30, 2026. The copyright holder for this preprint (which was not certified by peer review) is the author/funder, who has granted bioRxiv a license to display the preprint in perpetuity. It is made available under aCC-BY 4.0 International license.

Pearson correlation. For each pair of methods, we computed the Pearson correlation between their matrix-level summary scores across all matrices. High correlation indicates that the two methods rank drug pairs similarly; low correlation indicates that the methods capture fundamentally different aspects of the dose-response landscape or define additivity differently.

Sign disagreement rate. For each pair of methods, we computed the fraction of matrices for which exactly one method reports positive synergy (score > 0) while the other reports antagonism (score < 0). This captures qualitative disagreement: the methods not only rank differently but reach opposite conclusions about the direction of interaction.

Top-hit overlap. For each pair of methods, we identified the top $q = 5\%$ most synergistic matrices by each method's score, then computed the Jaccard index (intersection over union) between these hit sets. Low overlap means that the two methods would prioritize different drug pairs for experimental follow-up, with direct consequences for resource allocation in screening campaigns.

These summaries complement per-dose-point disagreement by quantifying instability at the level of global hit calling, which is the primary output of most synergy screening workflows.

7 Supplementary Note 1: software and reproducibility

The SIR software and all analysis code are available at `https://github.com/AsiaeeLab/SIR`. The repository contains the R source code implementing the SIR framework (isotonic regression, monotone-additive fitting, wild bootstrap testing), baseline method implementations (Bliss, HSA, Loewe, ZIP), visualization functions, and the complete set of scripts used to generate every figure and table in this manuscript.

All analyses are driven by YAML configuration files (e.g., `configs/default.yaml`) that specify transform parameters, bootstrap settings, and output directories, ensuring that results are fully reproducible from a single configuration. The constrained regression problems (isotonic and monotone-additive fits) are solved as convex quadratic programs using OSQP [2]. Intermediate results are stored as Parquet files, enabling exact reproduction of reported summary statistics without rerunning the full pipeline. The repository README provides step-by-step instructions for downloading the required datasets, restoring the R environment, and reproducing all results.

8 Supplementary Note 2: transform sensitivity

The null hypothesis of monotone additivity is defined on the scale determined by the response transform. Different transforms (identity on raw viability, logit, asinh, log) induce different notions of "additivity" and therefore different interaction surfaces and p-values. This is not a weakness of SIR but a fundamental property of any interaction test: the notion of "no interaction" depends on the scale.

For example, under the identity transform (raw viability), "additive" means that drug effects sum in viability units: if drug A reduces viability by 0.2 and drug B by 0.3, the combination is expected to reduce it by 0.5. Under the logit transform, "additive"

bioRxiv preprint doi: https://doi.org/10.64898/2026.02.05.704019; this version posted March 30, 2026. The copyright holder for this preprint (which was not certified by peer review) is the author/funder, who has granted bioRxiv a license to display the preprint in perpetuity. It is made available under aCC-BY 4.0 International license.

means effects sum on the log-odds scale, which compresses near the boundaries (0 and 1) and produces a different expected combination surface. A drug pair that significantly departs from additivity on one scale may be consistent with additivity on another. On a single example matrix, changing the transform from identity to logit changed the p-value from $p \approx 0.01$ to $p \approx 0.20$, not because the method is unstable, but because the two transforms define different null hypotheses and the data are consistent with additivity on the log-odds scale but not on the raw viability scale.

We recommend the logit link as a principled default for viability data because it maps $[0, 1]$ to $\mathbb{R}$, stabilizes variance near the boundaries, and defines additivity on a scale where equal increments correspond to equal log-odds changes in cell survival. When the appropriate scale is uncertain, we recommend reporting results under two or more transforms as a sensitivity analysis. The accompanying code repository provides a detailed exploration of transform effects on fitted surfaces, interaction maps, and bootstrap p-values.

9 NCI-ALMANAC replication

To assess generalizability beyond DrugCombDB, we repeated the baseline disagreement and pseudo-null calibration analyses on NCI-ALMANAC [3], a large-scale combination screen from the National Cancer Institute.

Baseline disagreement.

On a sample of 1,918 NCI-ALMANAC matrices with valid (non-NA) scores for all four baselines, Bliss and ZIP correlate near-perfectly ($r > 0.99$), while Loewe disagrees strongly with all other methods: sign disagreement rates of 38–43% and top-5% Jaccard overlaps as low as 0.06 (Supplementary Fig. 2). These patterns replicate and amplify the fragmentation observed on DrugCombDB (Fig. 1 of the main text). Notably, Loewe fails (non-finite output) on 97% of NCI-ALMANAC experiments, compared to 20.9% on DrugCombDB, reflecting the more challenging dose–response landscape of this dataset.

Pseudo-null calibration.

On 120 NCI-ALMANAC matrices subjected to the pseudo-null procedure (sign-flipping df-corrected residuals, $B = 200$), the resulting p-values are approximately uniform (Supplementary Fig. 3; median p-value 0.493), confirming proper calibration on an independent dataset.

References

[1] Wu, C. F. J. Jackknife, bootstrap and other resampling methods in regression analysis. *The Annals of Statistics* **14**, 1261–1295 (1986).

[2] Stellato, B., Banjac, G., Goulart, P., Bemporad, A. & Boyd, S. OSQP: an operator splitting solver for quadratic programs. *Mathematical Programming Computation* **12**, 637–672 (2020).

bioRxiv preprint doi: https://doi.org/10.64898/2026.02.05.704019; this version posted March 30, 2026. The copyright holder for this preprint (which was not certified by peer review) is the author/funder, who has granted bioRxiv a license to display the preprint in perpetuity. It is made available under aCC-BY 4.0 International license.

[3] Holbeck, S. L. *et al.* The National Cancer Institute ALMANAC: A comprehensive screening resource for the detection of anticancer drug pairs with enhanced therapeutic activity. *Cancer Research* **77**, 3564–3576 (2017).

bioRxiv preprint doi: https://doi.org/10.64898/2026.02.05.704019; this version posted March 30, 2026. The copyright holder for this preprint (which was not certified by peer review) is the author/funder, who has granted bioRxiv a license to display the preprint in perpetuity. It is made available under aCC-BY 4.0 International license.

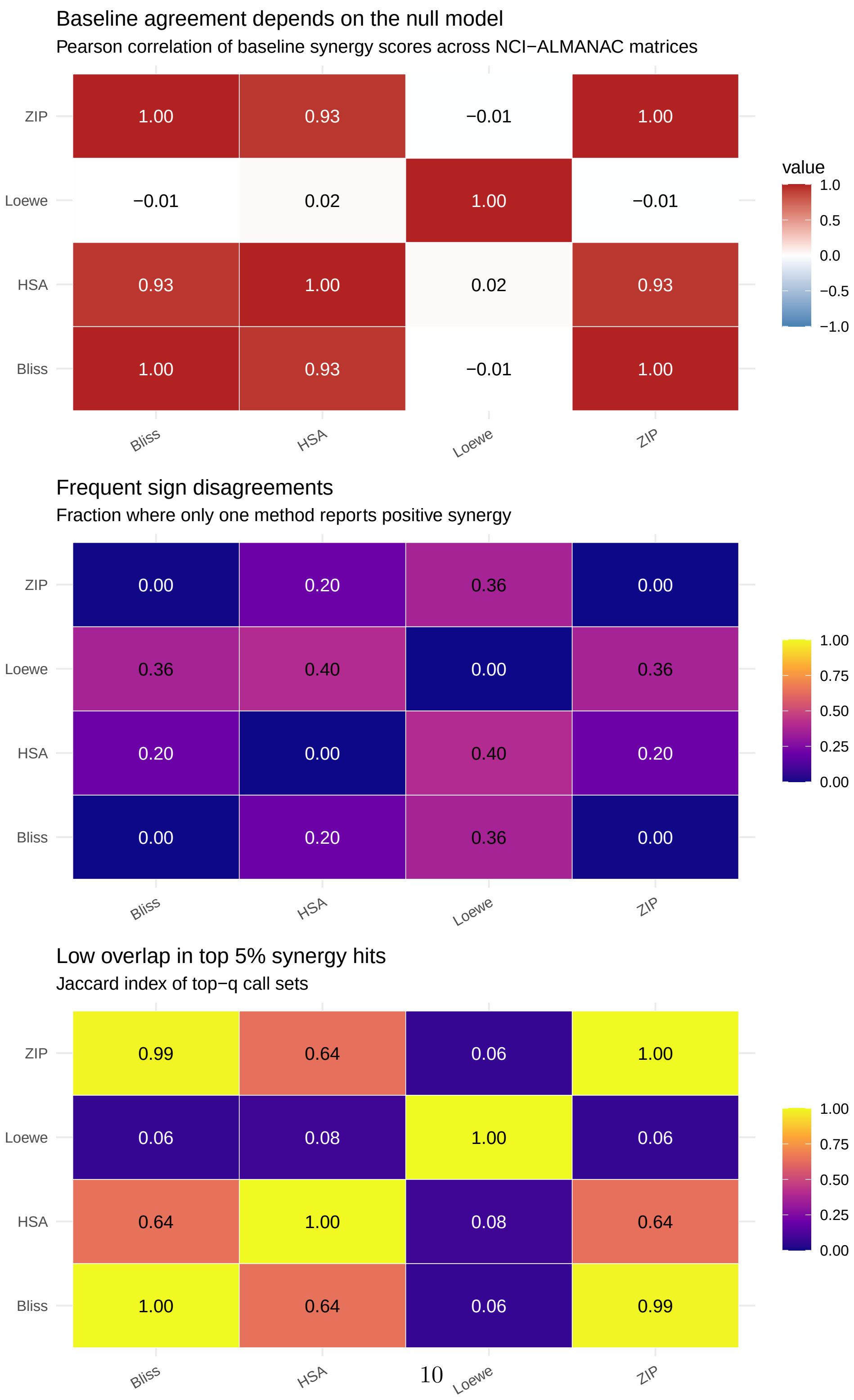

Supplementary Fig. 3 Baseline synergy score disagreement on NCI-ALMANAC ($n =$ 1,918 matrices with valid scores for all four methods). Top: Pearson correlations of matrix-level summary scores. Middle: fraction of matrices where only one method reports positive synergy. Bottom: Jaccard overlap of top 5% synergy calls. The disagreement pattern replicates and amplifies the DrugCombDB results (main text Fig. 1): Bliss and ZIP are near-identical ($r > 0.99$), while Loewe disagrees with all other methods ($r \approx 0$, Jaccard overlap 0.06), reflecting the fundamentally different dose-equivalence assumption.

bioRxiv preprint doi: https://doi.org/10.64898/2026.02.05.704019; this version posted March 30, 2026. The copyright holder for this preprint (which was not certified by peer review) is the author/funder, who has granted bioRxiv a license to display the preprint in perpetuity. It is made available under aCC-BY 4.0 International license.

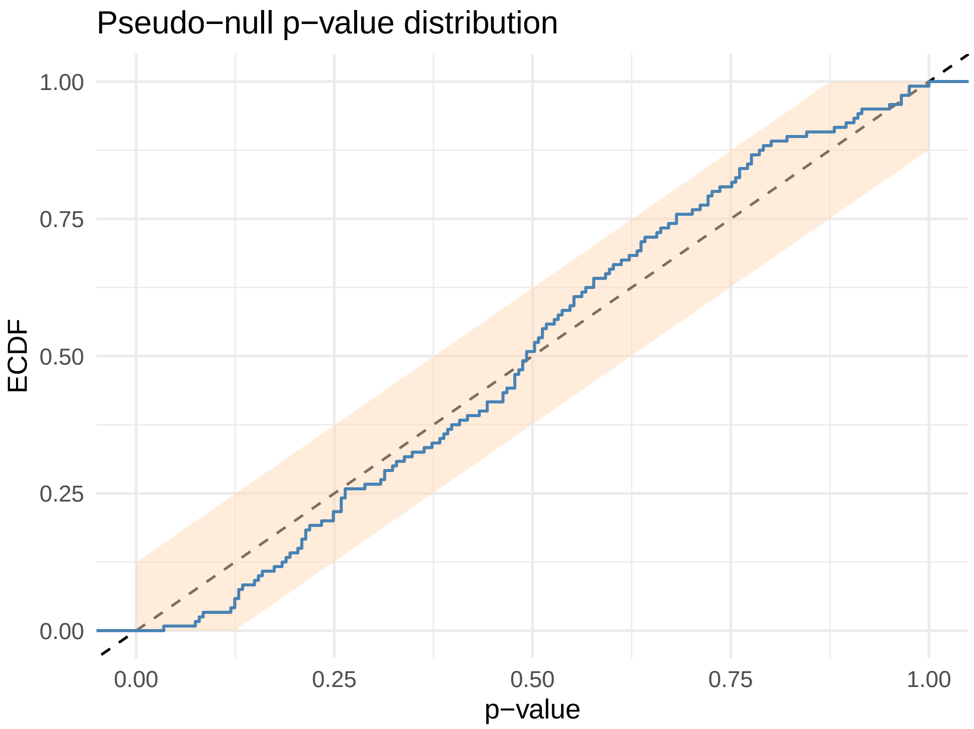

Supplementary Fig. 4 Pseudo-null p-value distribution on NCI-ALMANAC ($n = 120$ matrices; $B = 200$). P-values from the df-corrected wild bootstrap when data are generated under the fitted additive null by sign-flipping corrected residuals, so that no true interaction is present by construction. The dashed diagonal is the Uniform(0,1) reference; the shaded region is a 95% Dvoretzky–Kiefer–Wolfowitz band. The close agreement with the uniform diagonal (median p-value 0.493) confirms that SIR's calibration, demonstrated on DrugCombDB in the main text (Fig. 3), generalizes to an independent dataset with different experimental protocols and dose-response characteristics.
